# Laparoscopic ovum pick-up for *in vitro* embryo production from dairy bovine and buffalo calves

**DOI:** 10.21451/1984-3143-AR2018-0057

**Published:** 2018-08-16

**Authors:** Hernan Baldassarre, Vilceu Bordignon

**Affiliations:** Department of Animal Science, McGill University, Quebec, Canada.

**Keywords:** Holstein, buffalo, prepubertal, accelerated genetic gain, calf oocyte

## Abstract

Laparoscopic ovum pick-up (LOPU) conducted on bovine and buffalo calves of 2-6-month of age, followed by *in vitro* embryo production and transfer into synchronous adult recipients, is a powerful tool for accelerated genetic gain and early dissemination of top genetics. In its current state, the technology is characterized by higher oocyte recovery rates, lower oocyte-to-embryo yields, and similar pregnancy and term development rates compared with adult counterparts. Improvements in oocyte competence have been made in recent years mainly through gonadotropin stimulation protocols tailored for prepubertal donors. These advances have brought the technology to the point of been apt for commercial application. However, future research must focus on increasing the proportion of fully competent oocytes recovered from calves thereby further empowering the role this technology platform can play in programs for accelerated dissemination of superior genetics.

## Introduction

Laparoscopic Ovum Pick-Up (LOPU) was first described in 1974 by Snyder and Dukelow who were able to recover 6 oocytes from 21 follicles aspirated from a ewe under laparoscopic observation ( Snyder and Dukelow, 1974 ). However, the technology was not fully developed until the early 1990’s stimulated by the development of *in vitro* embryo production (IVEP) technologies. The initial target was the collection of oocytes from valuable females of species where ultrasound-guided ovum pick-up (OPU) was very difficult or not possible, due to animal size issues. In that sense, LOPU rapidly became, and remains as, the method of choice for the collection of immature oocytes for IVEP in sheep and goats ( Baldassarre *et al*., 1994 ; Stangl *et al*., 1999 ; Baldassarre *et al*., 2002 ; Cognie *et al*., 2004 ). Moreover, LOPU-IVEP has also been applied for oocyte collection in wild life species including deer and big felines ( Miller *et al*., 1990 ; Locatelli *et al*., 2006 ; Baldassarre *et al*., 2017 ).

Another application with high potential that was envisioned in the early 90’s was the collection of oocytes from early prepubertal heifers, i.e. several months before they would have grown enough to be eligible for standard OPU. Several publications showed high oocyte yields from bovine calves of 2-6 months of age stimulated with gonadotropins and subjected to LOPU. In some cases, the number of oocytes was substantially higher than what was recovered from adult cows ( Armstrong *et al*., 1992 ; Revel *et al*., 1995 ; Tervit, 1996 ; Baldassarre, 1998 ). However, this initial interest in developing the technology to produce “calves from calves” shortly disappeared for two main reasons. On the one hand, commercial application was negated by the fact that it was extremely difficult to predict the production phenotype of a female at the age of 2 months. Hence, selecting which candidates would be best donors had to be based on pedigree only, which has rather limited accuracy. On the other hand, all studies were showing that bovine calf oocytes were significantly less capable of developing into transferable embryos following *in vitro* maturation/fertilization and culture (IVM/F/C), compared with oocytes from adult cows ( Damiani *et al*., 1996 ; Duby *et al*., 1996 ). As a result, efforts to further develop this technology were practically abandoned for two decades.

More recently, the interest in developing the technology to a commercial level has resurged. First, with the advent of genomic marker technology it is now possible to better predict the production phenotype of dairy cattle from the moment they are born ( Hayes *et al*., 2009 ; Ponsart *et al*., 2013 ). To make use of that information in the most efficient way, breeders want to start reproducing the carriers of the best production genomes as soon as possible. Second, IVEP technologies have improved dramatically in the last three decades, including the possibility of freezing IVEP-blastocysts following direct transfer protocols, and obtaining pregnancy results comparable with those of *in vivo* produced embryos ( Moore and Hasler, 2017 ). Combined, especially in the highly competitive market of dairy genetics, the possibility of producing high quality embryos from elite females as early as 2 months of age has the potential of becoming a breeding target. Accelerated genetic gain by reducing generation intervals is a key objective. In addition, getting faster to the marketplace with new genetics is very attractive, especially for semen companies.

The present manuscript will summarize the state of the art as well as our most recent results working with Holstein cattle and Mediterranean buffalo calves subjected to repeated LOPU between the ages of 2-6 months.

## Step 1: Gonadotropin stimulation

Although females are born with all the oocytes they will produce through their lifetime, prepubertal animals have immature and non-functional hypothalamus-pituitary-ovarian axes, uncapable of supporting full follicular development and ovulation ( Sanchez and Smitz, 2012 ). However, waves of follicular activation and growth occur up to the antral follicle stages when they become gonadotropin-dependant. Early work clearly established that antral follicles from prepubertal ovaries can respond to exogenous gonadotropins. Furthermore, in most publications, the numbers of follicles aspirated, and oocytes recovered were significantly greater than the average for adults ( Armstrong *et al*., 1994 ; Duby *et al*., 1996 ; Tervit, 1996 ; Baldassarre, 1998 ).

Initially, short protocols in which gonadotropins were administered staring 36-48 before LOPU, were most popular because of their simplicity and effectiveness in generating large populations of follicles for aspiration. Some of these protocols used FSH alone and some used a combination of FSH and eCG. However, studies have shown that the developmental competence of oocytes increases with follicular size in heifers and cows ( Lonergan *et al*., 1994 ; Hendriksen *et al*., 2000 ; Kauffold *et al*., 2005b ). This is consistent with the knowledge that the oocyte acquires developmental competence as a result of the accumulation of critical molecules during follicular development, from the dormant primordial status to the pre-ovulatory stage. Some of those molecules are acquired by the oocyte’s own biosynthetic capacity and others are transferred from the cumulus granulosa cells through transzonal processes and gap junctions. Some molecules are necessary for oocyte growth, some are involved in the cross-talk with cumulus cells, some are needed later for oocyte maturation and fertilization, and some are needed even later to support embryo development until embryonic genome activation ( Blondin *et al*., 1997 ; Adhikari and Liu, 2009 ; Sanchez and Smitz, 2012 ). Hence, it is extremely important to provide a proper exogenous gonadotropin regime that will support follicular development and oocyte acquisition of developmental competence. In agreement with this concept, our work with Holstein calves of 2-6 months of age, showed higher rates of development to the blastocyst stage when oocytes were sourced from calves subjected to longer gonadotropin stimulation (3 days) compared with short (2 days) or non-stimulated, which was associated with higher proportion of larger follicles ( Table 1 ; Currin *et al*., 2017 ). Moreover, embryo development was significantly higher with oocytes recovered from larger (>5 mm) follicles (21%) compared with smaller follicles (11%, P < 0.05).

**Table 1:** Proportion of larger (>5mm) follicles in 2-6 month old Holstein calves stimulated with short, long or no gonadotropin treatments and in relations to cleavage and development rates following IVM/F/C.

	Follicles >5mm (%)	Cleavage (%)	Blastocyst (%)
No treatment	2.4^a^	66 ± 20^ab^	17 ± 9^a^
Short treatment	11.2^ab^	59 ± 22^a^	18 ± 15^a^
Long treatment	34.0^b^	73 ± 20^b^	37 ± 25^b^

Values in the same column with different script differ significantly (P < 0.05).

It is interesting to point out that the longer gonadotropin regimes used in our studies, resulted in 50-70% of larger follicles (>5mm in diameter). This allows speculating that there is room for further improvement in development yield by means of designing new hormonal regimes capable of increasing the proportion of larger follicles.

**Figure 1:**
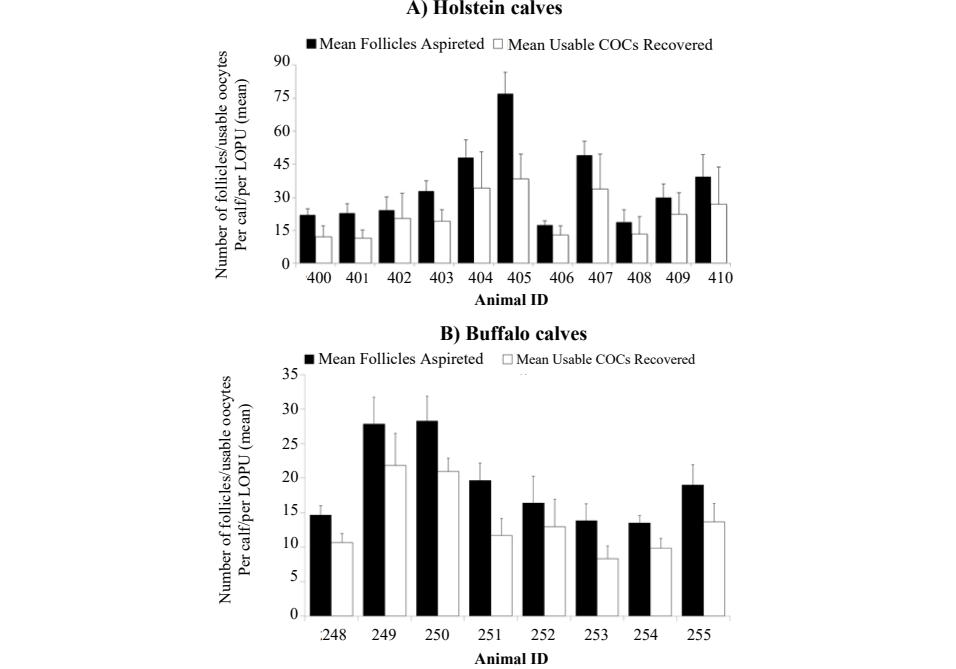
Individual variation in the mean number of follicles available for aspiration and usable COC’s recovered by LOPU in calves of 2-6 months of age. Bars represent the average from 6 LOPU procedures per each individual calf.

Notably, we observed a high degree of individual variation that was consistent throughout our studies with Holstein calves. In our most recent study ( Baldassarre *et al*., 2018 ), with a minimum of six LOPU procedures in a period of 3-4 months, the average number of usable oocytes recovered per Holstein calf/per LOPU was 22.2 ± 14, but it ranged from 38.2 ± 11 in the top calf to 12.7 ± 4 for the bottom one ( Fig. 1A ). Accordingly, the total number of oocytes collected per calf (sum of 6 LOPU) averaged 126.6 oocytes/calf, ranging from 229 in the top calf to 72 oocytes in the bottom one. Consistent with these results, working with Mediterranean buffalo calves of similar age, hormonal stimulation and number of procedures, the average number of usable oocytes recovered per calf/per LOPU was 16.2 ± 9, but ranging from 26.6 ± 6 to 10.07 ± 3 for the top and bottom calves, respectively. Moreover, the total number of oocytes collected per buffalo calf (sum of 6 LOPU) averaged 81 oocytes/calf, ranging from 130 to 50 oocytes in the top and bottom calves, respectively ( Fig. 1B ). In both species, it could be speculated that these results could have value as an early indicator of the ovarian response expectations one can have, if/when these animals were used as oocyte donors later in life as adults. Another aspect of individual variation worth mentioning was the timing of occurrence of their peak response, as assessed by number of follicles available for aspiration and oocytes recovered. Prior to our recently published work, there were no studies in which the same animals were collected repeatedly and frequently during the 2-6 months of age period. In the lack of such studies, when high responses were observed in calves hormonally stimulated and collected just once, one rational explanation was that it could result from accumulation of follicles from different growth waves at the antral stage, since the animals lack endogenous gonadotropin support for further follicle development. If this hypothesis was valid, most of our calves would have had their peak response on their first LOPU. However, what we observed in the Holstein calves was that only 10% had their peak at LOPU 1, while 36% had their peak at LOPU 2, 27% at LOPU 3, and the rest was scattered through LOPU 4, 5 and 6 ( Baldassarre *et al*., 2018 ). Similarly, 12% of Mediterranean buffalo calves peaked at LOPU 1, 37% at LOPU 2, and the rest scattered through LOPU 3 to 6. In summary, it remains unclear what are the mechanisms involved in the increased responsiveness to exogenous gonadotropins observed in prepubertal females.

Finally, age and number of previous procedures did not have significant effect with regards to follicular response and total oocytes recovered, when comparing calves of <100, 100 to 130, and >130 days of age.

## Step 2: Oocyte collection

LOPU is the procedure of choice for the collection of immature oocytes from medium sized animals that are too small to be collected by ultrasound-guided OPU (i.e. calves, sheep, goats, deer, etc.). In sheep and goats, it has been widely used to source oocytes for standard IVEP, for transgenic founder generation by pronuclear microinjection and for cloning by somatic cell nuclear transfer. In cattle and buffalo, it has been successfully used for the generation of embryos and offspring from oocytes collected from prepubertal females of 2-6 months of age. The procedure is minimally invasive and has shown to be very safe, specifically with no intraoperative complications nor sequels with potential for impact on the reproductive future of the animal. Moreover, all the calves that were used in our studies were subjected to LOPU 6 to 9 times in a 3-4 months period, and none of them had problems producing more embryos by OPU and/or getting pregnant later in their life. To facilitate ovarian visualization and prevent anesthesia complications (e.g. regurgitation), animals must be deprived from food and water for at least 24 and 12 hours, respectively. LOPU must be conducted under general anesthesia. Different anesthesia protocols can be used. In our most recent studies we induced anesthesia to allow intubation with a mixture composed of 0.05 mg/KBW xylazine, 2 mg/KBW ketamine and 0.1mg/KBW diazepam, administered intravenously, and maintained under anesthesia with 2% isoflurane. Once under, the calves are restrained on a cradled table in Trendelenburg position and the ventral area cranial to the udder is clipped and disinfected with 2% chlorhexidine followed by 10% iodine solution. Under laparoscopic observation, all follicles of ≥ 2mm diameter are aspirated using a 20G needle mounted on an acrylic pipette connected to a collection tube and a vacuum pump. The laparoscopic equipment consists of a 5mm/0° laparoscope, 3 trocar/cannula ports, an atraumatic grasping forceps, and a cabled light source. The grasping forceps is used for exposing the different surfaces of the ovaries to allow aspiration of all follicles of ≥2 mm, by pulling from the mesosalpinx in different directions. The vacuum pressure is adjusted to 50mmHg at the pump and 60 drops of media reaching the collection tube per minute, using a flow valve inserted in the vacuum tubing. The oocyte aspiration medium we use is Hepes-buffered Thyrode’s-lactate (TLH) supplemented with 10U/mL of heparin, 25μg/mL of gentamicin, and 0.1% polyvinyl alcohol. After all follicles are aspirated, the ovarian surface is rinsed with warm saline solution using a pipette introduced through one of the cannula ports. Once the procedure is completed, all instruments are removed, trocar incisions closed with a suture stitch or surgical glue, and animals are medicated with a preventative dose of antibiotic (e.g. 20 mg/KBW long acting oxytetracycline) and with an analgesic (e.g. 1mL/45 KBW flunixin meglumine), subcutaneously.

It is worth highlighting that, unlike what happens during OPU, during LOPU the ovarian stroma is never perforated by the needle, which only penetrates the follicular wall, hence resulting in little to no bleeding. Also, the rinsing of the ovarian surface with saline at the end of the procedure, thereby cleaning the ovary of any blood (which cannot be done by OPU), provides an additional layer of safety towards avoiding the potential for adhesions with negative impact on future fertility of the animals.

## Step 3: *In vitro* embryo production

Previously described standard bovine operating procedures and media were used in our studies for IVM/F/C to the blastocyst stage, in Holstein cattle and Mediterranean buffalo ( Landry *et al*., 2016 ). In general, this seems to be the common procedure from what is found in the literature. Indeed, most articles show little-to-no deviations in the procedures respect of those in use for oocytes from adult cows and heifers ( Revel *et al*., 1995 ; Armstrong *et al*., 1997 ; Khatir *et al*., 1998 ; Majerus *et al*., 2000 ; Taneja *et al*., 2000 ; Kauffold *et al*., 2005b ; Currin *et al*., 2017 ).

Fixing and staining of subsets of Holstein/Mediterranean calf-oocytes following IVM and IVF have allowed us identifying that ~80% of the calf oocytes can complete nuclear maturation. This suggests that this aspect of oocyte competence, i.e. the ability to resume meiosis and re-arrest at metaphase II with extrusion of the first polar body, may be adequate in calf-oocytes. However, the competence to undergo the cortical reaction following fertilization, a critical step for preventing polyspermy, is one aspect of oocyte competence that seems to be compromised. Lower number and delayed redistribution of cortical granules were observed by electron microscopy studies at the end of IVM, in bovine calf-oocytes respect of their cow counterparts ( Damiani *et al*., 1996 ). This deficiency negatively impacts on the oocyte’s ability to manage monospermic fertilization and synchronous pronuclear formation. Consistent with the above, we found polyspermy rates of 20-45% as assessed by fixing and staining subsets of presumptive zygotes 15-20h following IVF. The rate of polyspermy was unaffected by the gonadotropin stimulation protocol but was affected by donor age (older = better) and insemination dose (lower = better).

Overall, we obtained cleavage rates of 60-70% and the blastocyst yield around 14% of the total oocytes collected and >20% of those that cleaved. Development to the blastocyst stage was affected by gonadotropin treatment, with higher rates obtained for longer compared with shorter protocols, as well as for protocols combining FSH and eCG compared with FSH alone. This is consistent with previous studies in older animals ( Hendriksen *et al*., 2000 ; Machatkova *et al*., 2004 ) and non-stimulated bovine calves ( Kauffold *et al*., 2005a ), where oocytes collected from larger follicles were capable of higher rates of development to blastocyst.

In the case of oocytes from buffalo calves, only 50% of the oocytes were capable of nuclear maturation, indicating that further optimization of IVM conditions are necessary for increased IVEP efficiency. However, polyspermy rates (10-45%) were similar to those described above for Holstein calves and subject to the same variables (e.g. age, semen dose), and blastocyst yield was ~10%.

Age was also a factor with influence on *in vitro* developmental capacity. The Holstein development rates to the blastocyst stage doubled at >130 days compared with <100 days of age (19.8 vs. 9.5) and embryo quality was also superior in the older age group, based on mean cell numbers in blastocysts fixed and stained on day 7 (119.1 ± 47 vs. 91.5 ± 25). These results, however, need to be interpreted with consideration for the fact that the animals were subjected to repeated hormonal stimulation and oocyte collection between the youngest and the oldest age in the study. Consequently, it is not possible to separate the effect of age from the effect that multiple hormonal treatments may have had on the oocyte competence of prepubertal animals at later ages.

## Step 4: Embryo transfer

The developmental capacity of calf oocytes was further tested by transferring Day 7 blastocysts into adult recipients that were estrus synchronized. In the case of Holsteins, we transferred 21 blastocysts into an equal number of recipients, of which 13 became pregnant (62%). Of those 13 pregnant recipients, only 9 were allowed to remain pregnant and 100% of them carried their pregnancy to term. These results are comparable with those obtained with *in vitro* produced embryos in adult cows and a substantial component of the optimism for full development of the technology. While we still don’t understand entirely why only 10-20% of the oocytes recovered from Holstein calves are capable of development to blastocyst, it is very encouraging to see that those that do are capable of full development to term at similar rates as adult-derived blastocysts. In the case of Mediterranean buffalo, we had very limited access to adult animals, but 3 of 10 recipients transferred became pregnant (30%) and they should be calving in the next few months.

## Balance and future research

At current level of efficiency, if calves are collected by LOPU every 2 weeks between 2 and 6 months of age (total 8 LOPU), the expectation would be to obtain in average a total of 180 oocytes/Holstein calf and these should result in an average of 25 transferable blastocysts/calf. Following transfer into recipients, the expectation would be to produce at least 12 calves which would be born before or around the time that the mother reaches the age and weight to be bred to the first time. This is the true power of this technology, the ability to accelerate genetic gain by shortening of the generational interval and the possibility to reach the market faster with the new generation of elite genetics.

We believe the potential for further improving the efficiency of the technology is very realistic. Thirty years ago, during the early days of commercial IVEP in dairy cattle, expected results were one transferable blastocyst/Holstein cow per OPU. In current state of the art, the LOPU-IVEP technology applied to Holstein calves is already much more efficient. In addition, because of the larger number of oocytes per procedure that are collected per calf compared with cows, the potential for efficiency improvement is substantial.

We visualize two major areas of research with focus on increasing oocyte competence in young heifers. The first area is hormonal priming, i.e. better conditioning of the ovaries to stimulate increased intrafollicular acquisition of oocyte developmental competence. As mentioned earlier, the target is increasing the proportion (and size) of large follicles, since there are no doubts this is linked to oocyte competence. This could be achieved by means of modifying/extending the period of gonadotropin stimulation. Another road for exploration could be coasting, i.e. a period of FSH starvation following FSH stimulation and prior to oocyte collection, which is standard practice in adult cow OPU ( Nivet *et al*., 2012 ). However, this approach has yielded poor results when applied to a limited number of calves of <6 months of age ( Baldassarre *et al*., 2017 , McGill University, Quebec, Canada: Unpublished), which indicates that coasting procedures used in adult heifers and cows need to be adapted for the different hormonal environment of young calves.

The second area is the development of IVM protocols specially tailored for promoting acquisition of competence by calf oocytes. In this regard, strategies for delaying nuclear maturation and improving cytoplasmic maturation should be considered. Also, supplementation of IVM medium with e.g. growth factors, cytokines and embryokines have the potential for allowing increased accumulation of developmental competence-critical molecules in calf ooplasms.

## Conclusion

The LOPU-IVEP technology for 2-6-month-old calves is currently at commercial levels of efficiency and has substantial potential for doubling or even tripling its performance in the near future. Although some efforts in this direction may continue at the academic level, the development of the platform to its full capacity will require funding commitment from the larger commercial companies in the bovine and buffalo genetics and assisted reproduction fields. For them, the justification may come in the form of a simple question, a question of time: is it worth having those elite calves born earlier?
